# More than a Corepressor: The Role of CoREST Proteins in Neurodevelopment

**DOI:** 10.1523/ENEURO.0337-19.2020

**Published:** 2020-03-06

**Authors:** Simon Maksour, Lezanne Ooi, Mirella Dottori

**Affiliations:** 1 Illawarra Health and Medical Research Institute, Wollongong, New South Wales 2522, Australia; 2 School of Medicine, University of Wollongong, Wollongong, New South Wales 2522, Australia; 3 School of Chemistry and Molecular Bioscience, University of Wollongong, Wollongong, New South Wales 2522, Australia

**Keywords:** CoREST, differentiation, gene expression, neurodevelopment, REST, transcription factor

## Abstract

The molecular mechanisms governing normal neurodevelopment are tightly regulated by the action of transcription factors. Repressor element 1 (RE1) silencing transcription factor (REST) is widely documented as a regulator of neurogenesis that acts by recruiting corepressor proteins and repressing neuronal gene expression in non-neuronal cells. The REST corepressor 1 (CoREST1), CoREST2, and CoREST3 are best described for their role as part of the REST complex. However, recent evidence has shown the proteins have the ability to repress expression of distinct target genes in a REST-independent manner. These findings indicate that each CoREST paralogue may have distinct and critical roles in regulating neurodevelopment and are more than simply “REST corepressors,” whereby they act as independent repressors orchestrating biological processes during neurodevelopment.

## Significance Statement

The molecular mechanisms governing normal development of the brain are yet to be fully elucidated. The regulation of gene expression by transcription factors plays a significant role in the specification and maturation of neurons and glia. Repressor element 1 (RE1) silencing transcription factor (REST) has been well characterized as a transcriptional regulator of neurogenesis through the formation of a complex with the REST corepressor (CoREST) proteins. Recently, the CoREST protein family has been shown to independently target unique genes, have distinct expression patterns, and important REST-independent functions during neurodevelopment. Understanding the molecular mechanisms governed by the CoREST family will provide insight into the regulatory networks directing normal neurodevelopment.

## Introduction

Understanding the complex molecular mechanisms regulating gene expression in the brain is integral in providing insight into the processes that govern normal development, and conversely, are disrupted in neurologic diseases. The precise and tightly regulated differentiation of stem cells during embryogenesis and neurogenesis is essential for cells, tissues and organs to form and function properly. Transcription factors play an important role in regulating both pluripotency and cell differentiation by controlling expression patterns of genes critical for development (Boyer et al., 2005). One transcription factor that governs pluripotency and cell fate is repressor element 1 (RE1) silencing transcription factor (REST; also known as neuron restrictive silencer factor, NRSF). Through repressing the expression of target genes, REST regulates neurogenesis, neuronal differentiation and maturation (Paquette et al., 2000; Ballas et al., 2005; Gupta et al., 2009; Gao et al., 2011; Mandel et al., 2011), in addition to a playing role in neuroprotection (Lu et al., 2014; Song et al., 2016, 2017a). Dysfunction of REST and its corepressor proteins are hypothesized to cause disruption in gene regulatory networks, contributing to the pathophysiology of neurodegenerative conditions, including Alzheimer’s disease (AD; Lu et al., 2014; Ashton et al., 2017; Meyer et al., 2019), Huntington’s disease (HD; Zuccato et al., 2003, 2007; Conforti et al., 2013), Parkinson’s (Suo et al., 2015; Huang et al., 2019; Kawamura et al., 2019), and Prion disease (Song et al., 2016, 2017a,b).

Genome wide analysis revealed ∼2000 potential REST targets genes in the human genome (Bruce et al., 2004; Johnson et al., 2006). REST represses transcription by forming a complex with the REST corepressor 1 (CoREST1) and recruiting chromatin modifying enzymes to induce a condensed chromatin state. Two paralogues, CoREST2 or CoREST3, have also been shown to form a complex with REST (McGann et al., 2014; Jung et al., 2018). However, the importance of the CoREST proteins is only just emerging, as evidence suggests they have the ability to target unique genes, in a REST-independent manner, in various neural and glial cell types at different stages of development (Abrajano et al., 2009a, b; Wu et al., 2018). The expression profile, regulatory networks and function of the CoREST family in neurodevelopment is only partially defined. In this review we discuss what is currently understood about the role of the CoREST family in neurodevelopment and how these proteins have a broader spectrum than acting solely as “REST corepressors.”

## REST

REST was initially discovered in 1995 as an integral component of the central nervous system through its role as a master negative regulator of neuronal gene expression (Chong et al., 1995; Schoenherr and Anderson, 1995). REST is a member of the Kruppel-type zinc finger transcription factor family, containing eight GL1 Kruppel zinc fingers in the DNA binding domain (Chong et al., 1995; Palm et al., 1999). The binding domain allows REST to bind to its target genes through the highly conserved 21 base pair DNA sequence motif, known as the RE1 site (Chong et al., 1995; Schoenherr and Anderson, 1995). Chromatin immunoprecipitation-coupled with deep sequencing (ChIP-seq) experiments have identified REST to bind with ∼2000 genes within the human genome (Satoh et al., 2013; Rockowitz and Zheng, 2015) and 308 genes in neurons derived from human embryonic stem cells (ESCs) unique to the targets observed in ESCs (Satoh et al., 2013). Although the RE1 site is observed within a wide range of genes, it remains unclear whether REST interacts and represses expression at these sites *in vivo*.

### Role of REST in neurogenesis, neuroprotection, and neurodegeneration

REST is an important regulatory factor within the developing nervous system through repressing transcription of genes associated with neuronal differentiation and maturation (Tabuchi et al., 2002; Bruce et al., 2004; Ballas et al., 2005). Through the repression of neuronal genes REST regulates the switch between precursor cell specification and differentiation, with REST expression decreasing with development allowing for neuronal maturation (Su et al., 2004; Gao et al., 2011; Kim et al., 2015; Nechiporuk et al., 2016). REST mediated gene suppression is facilitated by the recruitment of two corepressor complexes, mammalian Swi-independent 3 (mSin3) and CoREST, that allow for the binding of chromatin-modifying enzymes (Ballas et al., 2005; Inui et al., 2017). In addition to its initial role in repressing neuronal genes in non-neuronal cells such as *Scn2a2* (encodes for Na_V_1.2), *Stmn2*, *Tubb3* (N-tubulin), *Grm2* (also known as GluR2), *Bdnf*, and *Calb1* (calbindin; Armisén et al., 2002; Kuwabara et al., 2004; Ballas et al., 2005), REST and its corepressor proteins have also been implicated in the regulation of other aspects of neurogenesis. For example, REST has been described to regulate phenotypic switches between neuronal subtypes, whereby increased levels of REST downregulate *Gad1* (encoding for GAD67) and reduce PV-positive GABAergic interneurons in mice (Singh et al., 2019). REST is also responsible for somatosensory neuronal remodeling in pain states, with genetic deletion of *Rest* in mice effectively preventing hyperalgesia (Zhang et al., 2019). REST regulates synaptic plasticity in the rat hippocampus through the timely developmental switch in synaptic NMDA receptors (NMDARs) through the repression of *Grin2b*, thus promoting NMDARs primarily composed of GluN2A subunits (Rodenas-Ruano et al., 2012). Other studies also provide evidence that REST plays a role in regulating the signaling cascades from neuronal insult to cell death. Under ischemic conditions REST levels are upregulated resulting in the suppression of GluR2 expression altering calcium permeability of CA1 neurons in the hippocampus thus hypothesized to affect influence neuronal survival (Calderone et al., 2003). Collectively, these studies have shown that the physiological role of REST is not only the repression of neuronal genes in non-neuronal cells but also governs broader aspects of neurogenesis and maintenance of mature neurons including regulating synaptic plasticity, neuronal remodeling and cell death.

REST also plays a role in neuroprotection, with aberrant expression or altered subcellular localization associated with a range of neurodegenerative diseases. In AD, there has been shown to be a decrease in REST expression in human cortical and hippocampal postmortem tissue. This finding was accompanied by a loss of nuclear REST and an upregulation of genes involved in cell death, Alzheimer’s pathology and an accelerated differentiation of neural progenitors (Lu et al., 2014; Meyer et al., 2019). In addition, a decline in REST plasma levels was associated with increasing severity of risk and impairment in patients with mild cognitive impairment and AD (Ashton et al., 2017). REST has been implicated in HD as mutant Huntingtin protein cannot sequester REST in the cytoplasm, leading to an increase in nuclear REST in striatal neurons and the repression of the REST target gene *BDNF*, contributing to an increased susceptibility to neuronal cell death (Zuccato et al., 2003, 2007; Conforti et al., 2013). REST was also shown to be an essential mediator of the neuroprotective function of the histone deacetylase (HDAC) inhibitor trichostatin A (TSA) Parkinson’s disease mouse model, as REST-deficient mice treated with TSA showed no improvement in dopaminergic neurotoxicity, TH and striatal BDNF levels and motor ability (Suo et al., 2015; Huang et al., 2019). The authors suggest that this effect is due to REST knock-out reducing adult neurogenesis and neural stem cell (NSC) survival (Huang et al., 2019). In human postmortem tissue, there is a loss of nuclear REST in aged dopaminergic neurons in Parkinson’s disease patients and an increased accumulation of REST in Lewy bodies and pale bodies, suggesting its sequestration in aggregates may diminish neuroprotective signaling (Kawamura et al., 2019). In an infectious model of Prion disease in hamsters and *in vitro* cell models, REST expression decreased and there was loss of nuclear REST. Overexpression of REST protected against the neurotoxic peptide PrP106-126, induced neuronal oxidative stress, mitochondrial damage, synaptic dysfunction, and neurofibrillary degeneration, potentially through the action of the Akt-mTOR and Wnt-β-catenin signaling pathways (Song et al., 2016, 2017a,b). Taken together, it is evident that REST plays a critical role in neurodevelopment, is required for normal aging and neuroprotection of the brain and exhibits region-specific and cell type-dependent effects in neurodegenerative diseases.

### REST-mediated gene repression

Chromatin is a complex critical for packaging DNA within the nucleus of a cell. The base unit of chromatin is a nucleosome which is composed of eight histones that are encircled by 147 base pairs of DNA. Histones have an unstructured N-terminal tail that allows for the regulation of transcription through changes in nucleosome-DNA interactions. Gene expression is regulated by transcription factors that activate or repress transcription through the stepwise recruitment of chromatin-modifying enzymes. Modifications of chromatin include acetylation (Allfrey et al., 1964), methylation (Allfrey et al., 1964), phosphorylation (Wei et al., 1999), sumoylation (Shiio and Eisenman, 2003), and ubiquitination (Sun and Allis, 2002).

REST exerts its repressive effects on target gene expression through recruiting two separate corepressor complexes, mSin3 and CoREST1 (formerly known as CoREST), which in turn facilitate the binding of chromatin-modifying enzymes (Ballas et al., 2005; Yu et al., 2011; Inui et al., 2017). mSin3A or mSin3B bind to the N terminus of REST and recruit HDAC1 and HDAC2 (Huang et al., 1999; Naruse et al., 1999; Grimes et al., 2000). The C terminus of REST binds the corepressor protein, CoREST1 (Barrios et al., 2014). Two paralogues, CoREST2 and CoREST3, have been found in humans and also form a complex with REST (McGann et al., 2014; Jung et al., 2018). However, their transcriptional activity and expression profile in the human brain remains largely unknown (Barrios et al., 2014; Sáez et al., 2015). Research suggests that each CoREST protein may play a different role in neurodevelopment via targeting unique genes in neural and glial cell types during development (Abrajano et al., 2009a,b, 2010).

**Figure 1.**
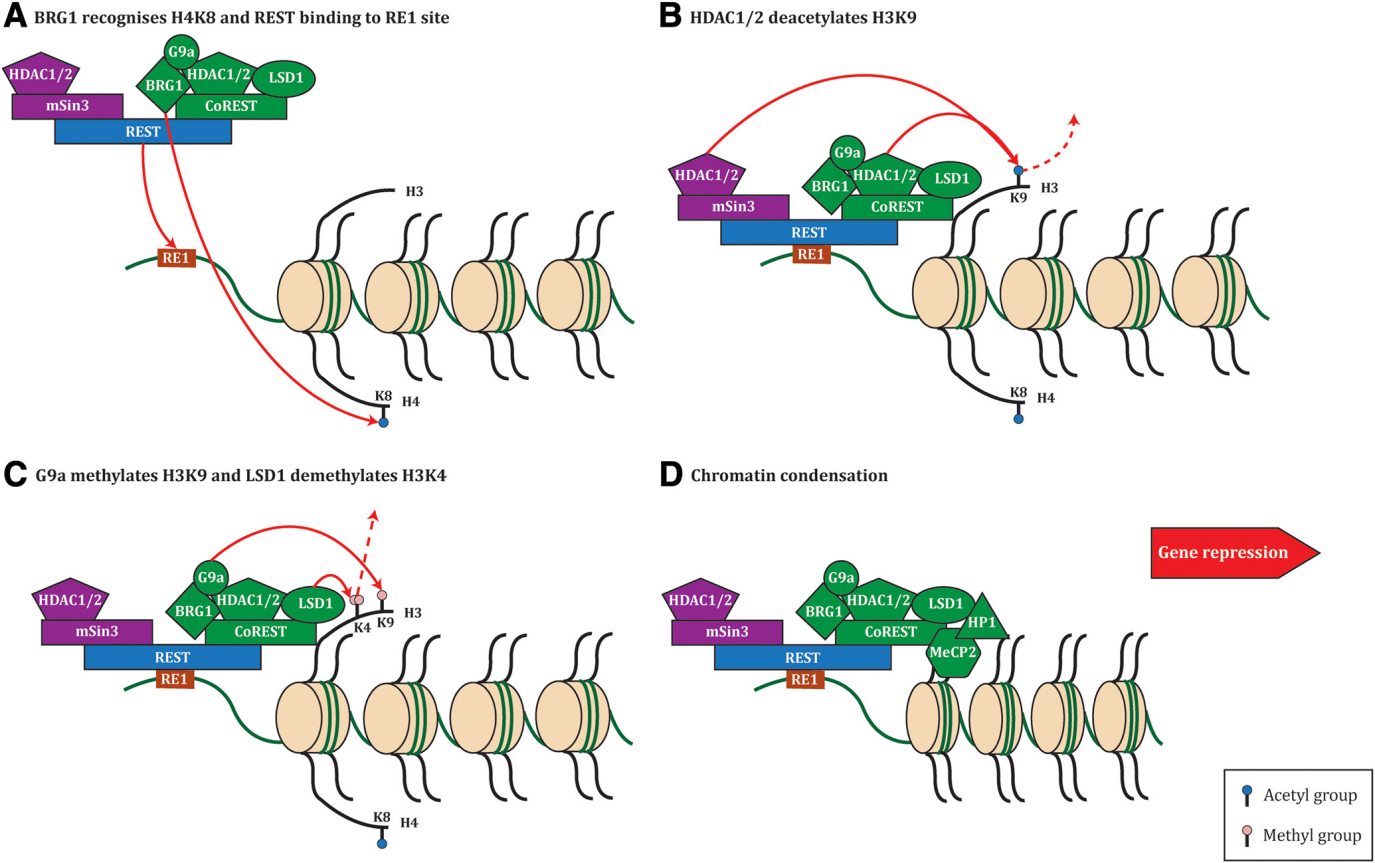
Schematic of REST-mediated gene repression. REST forms a complex with mSin3 (N-terminal) and CoREST (C-terminal) that in turn recruit an array of chromatin modifying enzymes. ***A***, Initially, REST binds to the RE1 site and is stabilized by the interaction between BRG1 and acetylated H4K8. ***B***, Following on, HDAC1/2 deacetylate H3K9. ***C***, G9a methylates H3K9 and LSD1 demethylates monomethylated or dimethylated H3K4. ***D***, Finally, chromatin is condensed via the recruitment of HP1 and MeCP2 to the high-affinity methylated H3K9, thus repressing gene expression.

In the complex with REST, CoREST proteins recruit complementary chromatin-modifying enzymes, including lysine-specific histone demethylase 1A (LSD1; also known as KDM1A), HDAC1/2, the H3K9 methyltransferase G9a, and the chromatin remodeling enzyme brahma-related gene-1 (BRG1) to target genes in order to regulate transcription (Battaglioli et al., 2002; Roopra et al., 2004; Lee et al., 2005; Ooi et al., 2006). To induce a repressive chromatin state, first BRG1 recognizes acetylated histone 4 lysine 8 (H4K8) and stabilizes REST binding to the RE1 site within target genes (Fig. 1*A*; Battaglioli et al., 2002). HDAC1/2 then deacetylates H3K9 (Fig. 1*B*), allowing for G9a to methylate H3K9 and LSD1 to demethylate monomethylated or dimethylated H3K4 (Fig. 1*C*; Tachibana et al., 2001; Roopra et al., 2004; Shi et al., 2005). The recruitment of heterochromatin protein 1 (HP1) and methyl CpG-binding protein 2 (MeCP2) to the high-affinity site of methylated H3K9 causes chromatin condensation and thus represses gene expression (Fig. 1*D*; Lunyak et al., 2002; Fuks et al., 2003). The stepwise activity of the REST complex is integral in the regulation of neurodevelopmental processes including neurogenesis (Gao et al., 2011), neuronal differentiation and maturation (Kim et al., 2015), synaptic plasticity (Rodenas-Ruano et al., 2012), and neuroprotection (Lu et al., 2014; Song et al., 2017b). Disruptions to REST-mediated gene repression are hypothesized to result in the breakdown of these key neuronal processes and contribute to the pathophysiology of neurodegenerative conditions.

## The CoREST Protein Family

The role of the CoREST family in neurodevelopment is less understood than those of REST. However, studies have indicated that CoREST proteins have distinct roles in neurogenesis, neuronal differentiation and maturation that are independent of REST. Despite their high sequence similarity in humans, evidence suggests each of the CoREST family members elicits unique functions at different stages of development (Yang et al., 2011; Wang et al., 2016; Jung et al., 2018). While the CoREST proteins appear to have independent roles, the exact function, target genes and expression pattern of each paralogue in neural and glial cells remains to be precisely defined. Biologically-relevant animal and cell-based models are essential for defining the molecular function of CoREST paralogues and providing insight into the mechanisms of neurodevelopment. To date, several different models, including rodent (Wang et al., 2016; Monaghan et al., 2017), stem cell (Yang et al., 2011), and established cell lines (Gómez et al., 2008), have been utilized to study the role of CoREST proteins in development. The current understanding of the CoREST family expression profile, target genes and functional roles in neurodevelopment are discussed in detail below.

### CoREST genes, transcripts, and protein structure

*REST corepressor 1* (*RCOR1*) is a 12-exon gene located on chromosome 14 that encodes CoREST1, a 53-kDa protein composed of 485 amino acids (Andrés et al., 1999). CoREST2 is a 58-kDa protein composed of 523 amino acids expressed by *REST corepressor 2* (*RCOR2*) which is a 13-exon gene located on chromosome 19 (Barrios et al., 2014). The final paralogue, CoREST3, is encoded by the 19-exon gene *REST corepressor 3* (*RCOR3*) located on chromosome 1, and is predicted to exist as four different splice variants. The variants are 48-, 50-, 56-, and 61-kDa proteins consisting of 436, 449, 495, and 553 amino acids, respectively (Barrios et al., 2014).

**Figure 2.**
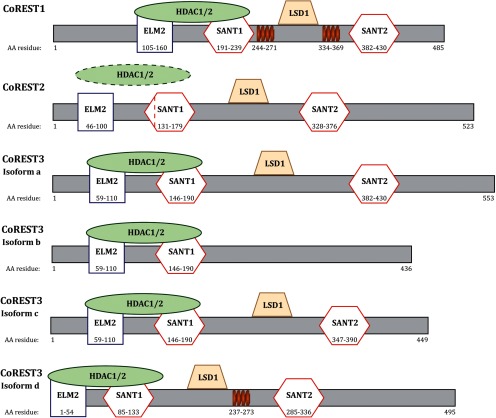
Structure of the CoREST proteins. Each CoREST paralogue contains an ELM2 domain and two SANT domains. The ELM2 and SANT1 domains are responsible for recruiting HDAC1/2. CoREST2 has a non-conserved leucine residue at 165 in the SANT1 domain resulting in impaired association with HDAC1/2. The linker domain between the SANT domains is responsible for binding with LSD1. CoREST3 isoform b lacks a SANT2 domain, resulting in impaired LSD1 recruitment and is responsible for the antagonistic action of the isoform. CoREST1 and CoREST3 isoform d both contain coiled-coil domains, represented by the orange coils. Information collated via UniProt Consortium (2018) and Marchler-Bauer et al. (2017).

CoREST proteins interact with the REST complex via a single zinc finger domain in the C-terminal half of REST (Andrés et al., 1999). A single point mutation resulted in abolished CoREST binding and transcriptional repression by the complex (Andrés et al., 1999). The function of the REST complex has been widely studied since discovery in 1995, with less known about the CoREST complex. Bioinformatics, structural analysis and immunoprecipitation assays of the CoREST family has provided insight into the components of the complex, how it interacts with DNA and potential mechanisms of epigenetic modifications to regulate gene expression. Each CoREST protein contains two Swi3, Ada2, N-CoR, TFIIIB (SANT) domains hypothesized to have a role in histone tail recognition and remodeling (Boyer et al., 2002, 2004; Lee et al., 2005; Shi et al., 2005) and a single Egl-27 and MTA homology 2 (ELM2) domain that acts as a protein-binding and potentially a DNA-binding site (Fig. 2; Solari et al., 1999; Lee et al., 2005; Barrios et al., 2014). The ELM2 and SANT1 domains are essential in recruiting HDAC1/2 (You et al., 2001; Ding et al., 2003; Lee et al., 2005). A nonconserved leucine at residue 165 in the SANT1 domain of CoREST2 results in impaired association with HDAC1/2 when compared with the other paralogues (Fig. 2, dotted red line; Barrios et al., 2014). The conserved linker domain between both SANT domains (Lee et al., 2005) or the SANT2 domain alone (Shi et al., 2005; Yang et al., 2006; Forneris et al., 2007) is responsible for interacting with LSD1. The SANT2 domain has been shown to mediate DNA binding (Yang et al., 2006; Pilotto et al., 2015). The shortest CoREST3 splice variant (isoform b; Fig. 2) only contains the SANT1 and ELM2 domain, limiting its ability to form a complex with LSD1, therefore reducing its transcriptional repressive capacity, and in some instances, resulting in the antagonism of CoREST1 (Barrios et al., 2014; Upadhyay et al., 2014). CoREST1 and CoREST3 isoform d are the only variants identified to have conserved coiled-coil domains (Fig. 2, orange coil; Marchler-Bauer et al., 2017; UniProt Consortium, 2018). Although the CoREST proteins share high sequence similarity, it may be their structural differences that result in a unique set of target genes and distinct functions in various cell types during neurodevelopment.

### CoREST-mediated gene repression

CoREST proteins are best documented for their transcriptional repression ability through their interaction with REST, however, new evidence demonstrates that they have the ability to repress unique target gene expression in a REST-independent manner. The CoREST proteins elicit their transcriptional repressive ability through the formation of a complex constituted of LSD1 and the HDACs HDAC1/2 in a 1:1:1 stoichiometry, known as the LSD1-CoREST-HDAC (LCH) complex (Barrios et al., 2014; Kalin et al., 2018). The significance of each CoREST paralogue during normal development remains a novel field, with continued research certain to provide insight into the regulatory mechanisms governing neurodevelopment.

**Figure 3.**
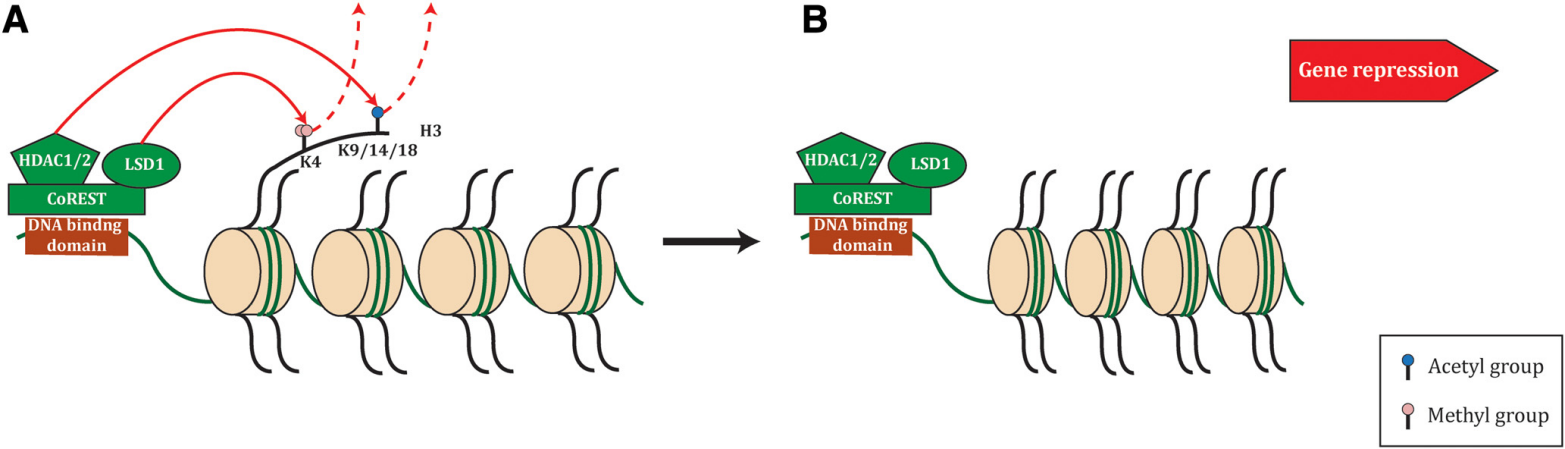
CoREST-mediated gene repression. CoREST forms a complex with HDAC1/2 and LSD1 to elicit transcriptional repression. ***A***, CoREST binds to DNA sites through the SANT2 domain. HDAC1/2 deacetylates multiple acetylated lysine marks on the H3 tail. LSD1 demethylates monomethylated or dimethylated H3K4. ***B***, The synergistic function of both chromatin modifying enzymes results in chromatin condensation thus repression of gene expression.

The transcriptional activity of the LCH complex is mediated by the synergistic effects of the HDAC and LSD1 enzymes (Kalin et al., 2018). The LCH complex binds to DNA through the SANT2 domain of CoREST which displaces the H3 tail (Yang et al., 2006; Pilotto et al., 2015). The DNA binding of the complex allows the histone tail to be available to the active sites of the chromatin modifying enzymes. HDAC1/2 deacetylates multiple lysine residues (K9, K14, and K18) on H3 tail, while LSD1 demethylates monomethylated or dimethylated H3K4 resulting in chromatin compaction and gene repression (Fig. 3; Pilotto et al., 2015; Wu et al., 2018). The LCH demethylase activity to H3K4 is significantly inhibited by H3K14 acetylation (Wu et al., 2018). Therefore, epigenetic regulation mediated by the LCH complex will be reduced when chromatin is marked by acetylation at Lys14, leading to a diminished repressive capacity towards genes that have an abundance of acetylated H3K14 in their promoter or enhancer region (Wu et al., 2018).

The variations in the CoREST protein structures are responsible for altered protein-protein interactions and thus differences in transcriptional repressive capacity (Barrios et al., 2014). Barrios and authors provide evidence that all three paralogues behave as transcriptional repressors through luciferase reporter assays. CoREST1 exhibited the highest transcriptional repressive capacity of the three paralogues. Nucleosomal demethylation assays demonstrated LSD1 could demethylate dimethylated H3K4 in free histones, but required CoREST1 for the demethylation of nucleosomes (Upadhyay et al., 2014). CoREST2 showed similar activity to CoREST1, however with a reduced efficiency. The reduced repressive activity of CoREST3 compared with CoREST1 was not a result of diminished interaction with LSD1 but potentially from a lower catalytic efficiency (Barrios et al., 2014). In erythroid cells, the shortest isoform of CoREST3 did not facilitate nucleosomal demethylation, instead acted as an antagonist competitively inhibiting CoREST1 activity (Upadhyay et al., 2014). CoREST-mediated nucleosomal demethylation was restored by appending the SANT2 domain from CoREST1 into CoREST3. The data suggests the antagonistic and inhibitory function of CoREST3 stems from the absence of the SANT2 domain observed in the short isoform (Upadhyay et al., 2014). It also indicates that the SANT2 domain is not only required for LSD1 recruitment but additionally is crucial in mediating LCH complex nucleosomal demethylation and thus is essential in CoREST-mediated gene repression activity independent to REST. HDAC activity and coimmunoprecipitation assays *in vitro* revealed CoREST2 to have reduced association with HDAC1/2 when compared with its paralogues due to a non-conserved leucine residue at 165 in the SANT1 domain (Barrios et al., 2014). CoREST2 mutants that had leucine 165 modified to a serine had similar repression activity as CoREST1 and CoREST3, indicating that CoREST2 mediates transcriptional repression in a HDAC-independent manner (Barrios et al., 2014). All CoREST proteins were confirmed to interact with all splice variants of LSD1 through coimmunoprecipitation assays, suggestive of a highly adaptable LCH complex (Sáez et al., 2015). Taken together, the versatility of the LCH complex is indicative of a wide range of novel gene targets that may be crucial in regulating neurodevelopment. There is a prominent void in the literature regarding the differences in transcriptional repression potency and activity between the REST-CoREST and the LCH complex. Further research is required to confirm the formation of the LCH complex *in vivo*, characterize the DNA sequence at the binding site of the complex and thereby identify the gene targets for each CoREST protein.

### Expression and subcellular distribution of the CoREST proteins during neurodevelopment

Current literature is suggestive of distinct expression profiles for each CoREST protein during neurodevelopment and in the mature brain depending on the cell type and developmental stage. The unique expression profile of each paralogue is suggestive of the formation of multiple LCH complexes, composed of a different CoREST protein core, with the potential to target a broad spectrum of target genes implicated in neurogenesis and neuronal maturation. Research has largely been based on animal studies, but provides valuable insight into the potential regulatory roles and functions the CoREST family may be involved in during neurodevelopment.

#### CoREST paralogues exhibit an age-dependent and region-specific expression pattern in the brain

Analysis of RNA-seq databases identified widespread expression of all CoREST paralogues, including the four splice variants of CoREST3 throughout rat adult brain tissue (Sáez et al., 2015). Sáez et al. (2015) used two models of differentiation, nerve-growth factor (NGF)-induced neuronal differentiation of PC12 cells and *in vitro* maturation of embryonic rat cortical neurons to document changes in mRNA and protein expression of the CoREST family in neuronal maturation. CoREST1 protein levels were reduced, but RNA levels for *Rcor1* remained similar throughout differentiation (Sáez et al., 2015). In addition, CoREST1 protein levels were shown to increase during embryonic development of the embryonic mouse reaching the highest levels at postnatal days 0 and 15, followed by a reduction in the aged mouse cortex (Fuentes et al., 2012). CoREST2 mRNA levels decreased during differentiation of both PC12 cells and rat cortical neurons (Sáez et al., 2015). Additionally, CoREST2 has been shown to be highly expressed in human and mouse ESCs (Yang et al., 2011), and mRNA and protein widely expressed across most cell types of the wild-type mouse cortex (Wang et al., 2016). CoREST2 expression assessed by Western blot analysis was shown to decrease in embryonic mice brains, indicating CoREST2 may function primarily during embryonic development (Wang et al., 2016). Relative to CoREST1 and CoREST2 significantly less is currently known about the expression profile of CoREST3 during neurodevelopment. Sáez et al. (2015) concluded that CoREST3 levels remain unaltered during neuronal differentiation of both PC12 cells and rat cortical neurons. CoREST3 was also shown to be expressed in rat hippocampal, cortical and whole brain extracts via Western blot analysis (Sáez et al., 2015). As the CoREST3 expression pattern remains to be defined, knock-down and overexpression studies targeting *RCOR3* will aid in identifying whether CoREST3 plays a role in regulating neuronal differentiation. Collectively, this data indicates a preliminary expression profile for the CoREST family, suggesting CoREST1 and CoREST2 levels decrease with maturation in certain brain regions, while CoREST3 expression remains unaltered in rat cortical neurogenesis. The cell types used in each model may be responsible for the variances observed in expression patterns. Further studies focusing on the expression of each CoREST paralogue during human neurodevelopment in different regions of the brain will provide insight into the functions of the CoREST family.

#### Differential subcellular localization of the CoREST family in different cell types

CoREST1 and REST protein expression and localization in different neuronal and glial subtypes were analyzed by immunocytochemistry and Western blotting in primary mouse neural cells (Abrajano et al., 2009a,b, 2010). Both exhibited nuclear expression in NSCs and intermediate progenitors, with expression in both the nucleus and cytoplasm of cholinergic, GABAergic, glutamatergic, and medium spiny neuron subtypes (Abrajano et al., 2009a, 2010). In glial cells, both REST and CoREST1 were expressed ubiquitously in the nucleus or cytoplasm of astrocytes and oligodendrocytes (Abrajano et al., 2009b). Immunohistochemistry of adult rat brain tissue revealed CoREST1 and CoREST2 to be expressed in the nucleus of both neurons and glial cells identified by colocalization with β-III tubulin and GFAP, respectively (Sáez et al., 2015). CoREST3 was identified to be expressed in hippocampal, cortical tissue and mouse glial culture, suggestive of expression in both neurons and glia cell types (Sáez et al., 2015). As CoREST3 expression was described to remain unchanged during differentiation it is unclear whether the protein elicits a function. Identification of the subcellular localization of the transcription factor will provide insight into whether it is expressed in nucleus and may be potentially regulating gene expression. Collectively, the expression of CoREST1 and CoREST2 predominantly in the nucleus of both neural and glia cells is suggestive that they may be repressing gene expression by binding to chromatin in these cell types.

#### CoREST2 expression changes throughout cell division

CoREST2 was predominantly expressed in the nucleus in cell types of the embryonic mouse brain, however, exhibited diverse subcellular localization at different stages of the cell cycle (Wang et al., 2016). Immunohistochemical analysis showed CoREST2 in the nucleus of radial glia cells during interphase and mainly localized in chromosomes during metaphase in the ventricular zone. During anaphase, CoREST2 was partially translocated in the space between two sets of separated chromosomes (Wang et al., 2016). These findings reflect a similar pattern to LSD1 during cell cycle progression in ESCs (Nair et al., 2012). Taken together, these findings are indicative that CoREST2 may be forming a transcriptional repressive complex with LSD1 during interphase and repressing genes required for cell division and maturation.

Collectively, the expression profile and subcellular localization of CoREST proteins suggest they have the potential to regulate gene expression in both neuronal and glial cell subtypes, however, further research is required to confirm the same subcellular localization in human cells and to identify the specific target genes being repressed, further elucidating the functional roles of the CoREST family. Taken together, these findings indicate that the formation of multiple LCH complexes composed of a different CoREST protein core would broaden the genes targeted during neurodevelopment and may elicit various functions across neural and glial cell populations.

### CoREST proteins target unique genes compared with REST

REST-mediated gene repression through the formation of a complex with one of the CoREST proteins is expected to target ∼2000 RE1-site containing genes throughout the human genome, many essential for neuronal development (Bruce et al., 2004; Satoh et al., 2013). Research has defined a mechanism for CoREST proteins to act as transcriptional repressors independent to REST, targeting many distinct genes responsible for the modulation of neuronal and glial cell specification, maintenance, and maturation (Abrajano et al., 2009a, b, 2010; Yu et al., 2011). Genome-wide ChIP-seq in mouse ESCs investigating the binding of REST and its cofactors to sites on the genome identified CoREST1 to have 84 peaks with 61 overlapping with REST, CoREST2 to have 459 genomic binding sites and only 43 overlap with REST and CoREST3 to have 3744 peaks and 885 overlap with REST (Yu et al., 2011). Further work is required to identify whether the CoREST proteins binding directly to DNA, the cofactors recruited to the genomic binding site and whether the genes are functionally repressed by the activity of the complex. Through ChIP-on-chip analysis in mouse NSCs, CoREST1 was shown to bind to a broader range of genes (1820 genes) compared with REST (322 genes; Abrajano et al., 2010). Of these genes only 126 were targets of both REST and CoREST1. CoREST1 was identified to target a significantly greater percentage of genes involved in pluripotency such as NANOG/OCT4/SOX2 network compared with REST (79 compared with eight genes, respectively; Abrajano et al., 2010). Suggesting CoREST1 has a widespread role in regulating NSC gene networks that is unique to REST. Taken together, both REST and CoREST1 play a role in regulating the switch between NSC self-renewal and neural lineage specification, differentiation, and maturation. Among the genes targeted by REST, 72% contain known RE1 sites, whereas only 41% genes targeted by CoREST1 contain known RE1 sites, indicating CoREST1 may repress transcription at additional sites of DNA (Abrajano et al., 2010). In cholinergic, GABAergic, glutamatergic and medium spiny neurons, REST bound to 622, 587, 481, and 477 distinct genes, and CoREST1 bound 600, 814, 266, and 967 unique target genes, respectively (Abrajano et al., 2009a). Additionally, 3178 REST and 4060 CoREST1 target genes were observed in the two glial cell types, astrocytes and oligodendrocytes (Abrajano et al., 2009b). REST bound to 287 genes specific to astrocytes and 1365 genes specific to oligodendrocytes. CoREST1 was identified to interact with 40 unique targets in astrocytes and 963 genes in oligodendrocytes (Abrajano et al., 2009b). These studies have shown that REST and CoREST1 have the potential to regulate neuronal and glial differentiation, specification, and maintenance via the genes they target. Overall, these findings are suggestive that CoREST1 has a broad, cell type-specific role in neurodevelopment that is distinct and complementary to REST. The DNA sequence each CoREST paralogue targets, the complex formed at these sites and the gene networks regulated are yet to be defined. Further ChIP-on-chip studies with high resolution whole-genome approaches in human tissue will provide insight into the unique and interrelated regulatory networks of REST and CoREST paralogues.

### Functional roles of the CoREST family in neurodevelopment

The molecular mechanisms governing normal development of the brain are yet to be fully elucidated. The regulation of gene expression by transcription factors plays a significant role in the specification and maturation of neurons. Of the three paralogues, CoREST1 has been the most widely studied and is best understood for its role in REST-mediated gene repression. However, recent evidence has shown the CoREST paralogues interact with LSD1 and HDAC1/2 independently of REST and contribute to gene repression (Barrios et al., 2014; Pilotto et al., 2015; Wu et al., 2018). These studies have shifted our understanding that CoREST family act solely as REST corepressors but also have distinct and essential roles in regulating neurodevelopment. The known functions of the CoREST protein family in neurodevelopment is summarized in Table 1. Current knowledge regarding the role of the CoREST proteins has largely been based off animal studies. Rockowitz and Zheng (2015) showed that REST target sites do not completely overlap between human and mouse genomes, with human ESCs having twice as many REST sites as mouse ESCs via ChIP-seq analysis (*n* = 8199 vs *n* = 4107). From these findings it can be hypothesized that there may also be differences in the genes targeted by each CoREST protein between species. Continued research focusing on the action of the CoREST family will provide insight into the regulatory networks orchestrating neurodevelopment.

It is becoming evident that the CoREST family have unique functions independent to REST, in addition to having distinct roles for each paralogue. Monaghan et al. (2017) showed that *Rcor1/Rcor2* knock-out mice had severe deficits in neuronal and glial cell differentiation and a concomitant increase in *Rest* mRNA levels. Normalization of *Rest* levels fully restored one of the seven targets that was down regulated (*Celsr3*), the other transcripts were only partially restored (*Chrnb2*, *Trim67*, and *Unc13a*) whereas the remaining three were not rescued (*Fam65b*, *Gad2*, and *Scrt1*). These results indicate that Rcor1 and Rcor2 regulate the switch between proliferation and differentiation in the developing mouse brain in a predominantly Rest-independent manner. In addition, Fuentes et al. (2012) showed *Rcor1* knock-down resulted in impaired radial migration of cortical pyramidal neurons in the developing mouse cortex. To confirm the phenotype was mediated by CoREST1, the authors showed overexpression of CoREST1 with a mutated N terminus, to hinder association with Rest, could rescue the migration of neurons in the cerebral cortex. In addition, shRNA knock-down of *Rest* via electroporation at embryonic day 14 showed no differences in migration when compared with control. These results suggest that CoREST1 regulates pyramidal neuron development independent to Rest in the developing mouse brain. The CoREST paralogues have been shown to have distinct roles in the regulation of pluripotency independent to each other. Overexpression of *RCOR2*, but not *RCOR1*, was successful in the reprogramming of induced pluripotent stem cells (Yang et al., 2011). In chicken primordial germ cells, the knock-down of *RCOR3* resulted in the upregulation of the pluripotency regulator NANOG, whereas siRNA knock-down of *RCOR1* and other chromatin modifying enzymes known to form a complex with REST showed no significant changes in NANOG expression (Jung et al., 2018). Collectively, these studies show that the CoREST family have critical roles during neurodevelopment, that are independent to REST and may have compensating or distinct functions to each paralogue. Further ChIP-on-chip studies with high-resolution whole genome approaches are required to identify the binding sites of each CoREST protein in conjunction with knock-down and overexpression studies to identify the specific pathways and networks regulated, and thus deepen our understanding of the epigenetic mechanisms that govern neurodevelopment.

Our knowledge of the functional roles of the CoREST protein family stems from a heavy reliance on animal models, with the exemption of the study completed by Yang et al. (2011) who was successful in overexpressing *RCOR2* to reprogram human stem cells and Gómez et al. (2008) that investigated CoREST1-mediated regulation of the heat shock response in the human embryonic kidney cell line, HEK293. As previously stated, it has been shown that the targets of REST do not overlap between species (Rockowitz and Zheng, 2015), with the same hypothesized for the CoREST paralogues. Thus, human models of neurogenesis, such as human pluripotent stem cells, should be employed to further interrogate the molecular mechanisms regulated by the CoREST family.

**Table 1:** Summary of characterized functional roles of the CoREST family in neurodevelopment

	CoREST protein involved	Functional role	Species	References
Regulation of pluripotency	CoREST2	*Rcor2* knock-down resulted in reduced proliferation and impaired pluripotency; the overexpression of CoREST2, together with Oct3/4, Klf4, c-Myc, was successfully used to replace Sox2 in the generation of mouse and human induced pluripotent stem cells	Mouse and human	Yang et al. (2011)
	CoREST3	*RCOR3* knock-down resulted in significant upregulation of *NANOG* and enriched acetylated H3K9 residue on the REST binding site in the *NANOG* promoter region; indicating CoREST3 regulates *NANOG* expression through the formation of a complex with REST and the deacetylation of the *NANOG* promoter region	Chicken	Jung et al. (2018)
Regulation of neuronal differentiation and maturation	CoREST1	*Rcor1* knock-down resulted in impaired radial migration of cortical pyramidal neurons in the developing cerebral cortex; *Rcor1* knock-down cells exhibited delayed migration, remained in the ventricular zone and expressed Sox2 and Tbr2, suggesting the cells had not differentiated from precursor lineages	Mouse	Fuentes et al. (2012)
	CoREST2	*Rcor2* conditional knock-out (*Rcor2*^cko^) mice had significantly reduced brain sizes, cortical thickness, and structural abnormalities of the brain layers; *Rcor2*^cko^ mice had reduced numbers of neuronal progenitors and neurons, and increased cell death; the gene knock-out (KO) mice showed significant upregulation of ventral markers and decrease in cortical markers, suggesting CoREST2 regulates the sonic hedgehog signaling pathway	Mouse	Wang et al. (2016)
	CoREST1 and CoREST2	The individual gene knock-out (KO) mice were indistinguishable to the control cohort, combined deletion resulted in severe brain phenotypes and death; *Rcor1/2* KO mice had an increased population of proliferating cells, suggesting these mice lacked the mechanism to differentiate precursors into postmitotic neurons and mature oligodendrocytes; CoREST1 and CoREST2 are hypothesized to elicit this function through the formation of a complex with insulinoma-associated 1	Mouse	Monaghan et al. (2017)
Regulation of neuroinflammation	CoREST1	CoREST1 interacts with the promoter of *hsp70*, a gene that encodes heat shock protein 70 (Hsp70); through this interaction, CoREST1 represses both HSF1-dependent and heat-shock-dependent transcriptional activation of *hsp70*; *RCOR1* knock-down resulted in loss of Hsp70 repression, inducing the heat shock response	Human	Gómez et al. (2008)
	CoREST2	*Rcor2* expression was shown to decrease in an aging mouse model, accompanied by an increase in proinflammatory markers; *Rcor2* knock-down further increased inflammatory marker expression	Mouse	Alvarez-López et al. (2014)

## The CoREST complex as a potential target for therapeutics

Aberrant expression or subcellular localization of REST contributes to the disruption of gene regulatory networks and is associated with the pathophysiology of neurodegenerative conditions. It has been hypothesized that targeting REST may help protect from the progression of these conditions. For example, in Huntington’s disease (HD), the accumulation of nuclear REST in medium spiny neurons of the striatum results in enhanced repression of *BDNF* transcription contributing to an increased susceptibility to neuronal loss (Zuccato et al., 2003). A potential therapeutic for HD pathogenesis is targeting splicing of REST using antisense oligonucleotides *in vitro* to reduce the accumulation of nuclear REST (Chen et al., 2017). However, as REST targets the RE1 site in ∼2000 genes in the human genome, the modulation of REST remains a difficult target as it has the potential to have multiple downstream effects. An alternative is to target the LCH complex, for example, using corin, a derivative of a synthesized compound derived from the HDAC1 inhibitor (entinostat) and the LSD1 inhibitor (tranylcypromine analogue) to simultaneously target both components of the LCH complex (Kalin et al., 2018). The dual-hybrid agent has been successfully used as a potential therapeutic in cancer studies by reducing the proliferation of mouse melanoma cells through blocking the active sites of HDAC1 and LSD1 of the LCH complex (Kalin et al., 2018). Synaptic dysfunction is a common in many neurodegenerative diseases including AD, with HDACs involved in regulating synaptogenesis and synaptic plasticity (Fuller et al., 2019). HDAC inhibitors have been trialed to improve synaptic growth and function, but have been limited due to their off-target effects and dose-limiting hematologic toxicities. Fuller et al. (2019) used the CoREST-selective HDAC inhibitor Rodin-A in a mouse model and were successful in increasing spine density, expression of synaptic proteins and long-term potentiation at suitable doses to allow for chronic treatment. This study has shown that selectively targeting the CoREST complex, and not the Sin3, NCoR, and NuRD complexes, offers a promising therapeutic for synaptopathies and that the CoREST complex is a key regulator of synaptic growth and function (Fuller et al., 2019).

The regulatory mechanisms governing CoREST protein expression and function remain largely unknown. Sáez et al. (2018) have described a possible mode of regulation of the CoREST proteins via the interaction with protein inhibitor of activated STAT (PIASy) and SUMOylation which may control cell fate determination during development. Abrajano et al. (2010) proposed a mechanism in which CoREST1 regulates its own expression by binding to the gene *Senp1*, which encodes for a small ubiquitin-like modifier (SUMO)-specific protease and has been previously shown to inhibit CoREST1 activity (Muraoka et al., 2008). Future investigation into the regulatory networks of each CoREST paralogue will be crucial in understanding the processes of neurodevelopment and may provide potential therapeutic targets for neurodegenerative conditions.

## Summary

In summary, REST has been well documented for its function in neuronal differentiation and maturation, with new evidence emerging of a potential neuroprotective role in neurodegeneration. More focus needs to be dedicated to the CoREST family, as the importance of CoREST-mediated gene repression during neurodevelopment is continuing to grow in the field. It is clear that there are differences in the target genes between CoREST1 and REST in different neuronal and glial cell types, suggesting that each paralogue may play distinct and important roles in neurodevelopment. Future studies focused on the target genes, extensive expression profile and regulatory networks of the CoREST paralogues in different human neural and glial cell types will deepen our understanding of the tightly regulated molecular mechanisms of neurogenesis and normal brain development. It is clear that CoREST proteins are essential for proper neurodevelopment and dysfunction of these regulatory mechanisms are potentially linked to the progression of neurodegenerative conditions. Therefore, the CoREST family have a broader function outside of acting solely as a REST corepressor but are also independent and critical regulators of neurodevelopment.

## Synthesis

Reviewing Editor: Orly Reiner, Weizmann Institute of Science Decisions are customarily a result of the Reviewing Editor and the peer reviewers coming together and discussing their recommendations until a consensus is reached. When revisions are invited, a fact-based synthesis statement explaining their decision and outlining what is needed to prepare a revision will be listed below. The following reviewer(s) agreed to reveal their identity: Emily Barton, Lindsay De Biase.

This review manuscript highlights the independent functions of the CoREST protein family. Further understanding of the independent mechanisms of these transcription factors may provide insight into the development and progression of neurodevelopmental disorders.

The present review covers an interesting topic that has not received much attention in the literature, namely, potential REST-independent functions of CoREST proteins. The review shows potential but falls short of really delving into and fleshing out potential REST-independent functional roles for CoREST proteins. The major comments are largely focused around this idea - that not enough attention is given to the functional implications of studies that have looked at CoREST and what is needed to move the field forward. The rest of the comments are mainly small suggestions for improving clarity of the manuscript.

Reviewer #1:

This is a well-researched review paper that highlights a gap in knowledge of the independent functions of the CoREST protein family. Table 1 specifically is a very helpful summary of major findings from this review.

In the introduction, there is an over-reliance on citing other review articles instead of the primary research. However, this is not the case in the rest of the paper.

The introduction to section 3 has no citations, although, it mentions evidence, animal models, and cell models used to study the effects of CoREST dysfunction. It can be assumed the information provided there is broad and will be expounded later in the section with citations, however, a few substantiating citations may be beneficial.

There are some minor grammatical errors in section 4 lines 5-6 with incorrect or missing words.

Overall, this is a good review of the functions of the CoREST proteins in neurodevelopment.

Reviewer #2

Major comments:

1) The title leads us to believe that the meat of the review will be about functional roles of CoREST proteins in neurodevelopment, and more specifically, potential REST-independent functional roles of CoREST proteins. But the functional roles of REST and CoREST actually get relatively little treatment and elaboration compared to information about their expression patterns and composition of repressive complexes that they form etc.

a. In the introduction, functional roles of REST are summarized (“Through repressing the expression of target genes, REST regulates neurogenesis, neuronal differentiation and maturation (as reviewed in Ooi and Wood, 2007), in addition to a playing role in neuroprotection (as reviewed in Zhao et al. 2017). Dysfunction of REST and its corepressor proteins are hypothesised to cause disruption in gene regulatory networks, contributing to the pathophysiology of neurodegenerative conditions (as reviewed in Mampay and Sheridan 2019).” But no additional information is provided about the evidence for such functional roles and whether similar mechanisms are at operation in all these different contexts. In addition to citing the reviews, the authors should give us some summary of evidence suggesting these functions and the mechanisms involved. Is the mechanism exclusively transcriptional repression? Are different sets of genes targeted in each of these contexts and, if so, how is such specificity thought to be achieved?

b. Second paragraph in section 2, the authors say that REST represses transcription of genes associated with neuronal differentiation and maturation. But what are the consequences of this? That REST acts overall to promote maintenance of progenitor cell status? That REST regulates the timing of switches between neurogenesis and gliogenesis? That REST regulates the speed and timing of neuronal differentiation after cells have been specified?

c. Second paragraph in section 2: the authors tell us that REST and its co repressors have been shown to regulate neurogenesis, shape neuroprotection, and contribute to cancer and neurodenegerative disease when aberrantly expressed. But again they don’t discuss at all the evidence for these proposed functional roles and the potential mechanisms at work here. This is critical if we want to go on to decide whether CoREST proteins independently exert functional roles that are similar to or distinct from those that occur in concert with REST.

2) Section 3.5 “Functional roles of the CoREST family during neurodevelopment,” what should be the meat of the review, is packaged into a table. This should be the longest section of the review with a careful analysis of what exactly has been done to suggest that CoREST proteins can shape development independently of REST. What experimental approaches were used? What were the implications of these studies? What are the limitations of these studies and what is needed to advance our understanding of REST-independent CoREST function? Do we need specific mouse models that haven’t been made for example? The authors say in a number of places that more research is needed, but it would be helpful for them to indicate what are THE most critical gaps in our knowledge at this point and what would be key experiments that could fill these gaps. It’s fine to have a table that summarizes key studies, but this should NOT take the place of a careful analysis and discussion of current evidence and gaps in the literature.

3) Last two sentences of section 2.1 indicate that the stepwise activity of the REST complex influences neurogenesis, synaptic plasticity, and neuroprotection and that disruptions of this process contribute to neurodegenerative conditions. Again, since the title, significance statement and introduction indicate that we will be learning about the functions of REST and CoREST, it would be helpful to elaborate here on the what the evidence is for involvement in each of these functions. Is the same stepwise activity of the REST complex required for each of these functional roles? Are the target binding sites and genes the same or different for each of these proposed functional roles?

4) Section 3.1 goes into great detail about how CoREST proteins recruit other players to the complex. But if we want to learn more about potential roles for CoREST proteins independent of REST, we need different / additional information. What mediates the interactions of CoREST with REST and what potential mechanisms would determine when this interaction occurs and under what circumstances this interaction doesn’t occur? When CoREST proteins are acting independenty of REST - do they still need the interactions with all the other complex members to function?

5) Section 3.2 - emphasize that what is being presented is the mechanism by which CoREST proteins are thought to exert their REST-independent activity. Help the reader who may not be directly in the field by drawing some comparisons to the most important distinctions between REST-CoREST mediated transcriptional repression and CoREST mediated transcriptional repression. The Sant2 domain is mentioned as mediating DNA binding - is this absolutely necessary for CoREST LCH complex action on DNA independent of REST? What happens if this domain is mutated in CoREST proteins? What genes contain binding sites for this Sant2 domain and does this account for different genes being targeted by REST-CoREST versus CoREST LCH compex? How do REST-CoREST and CoREST LCH complex transcriptional repression compare in terms of potency and permanence?

6) When discussing the various genes targeted by REST and CoREST in section 3.4, it’s not clear how much overlap there is in the genes targeted by each. Are the genes targeted by REST just a subset of those targeted by CoREST or are they completely different genes? What are the broad functional families into which REST versus CoREST alone targeted genes? What does this suggest about functional roles of REST-CoREST complex versus the CoREST LCH complex? How much overlap is there in the genes targeted by CoREST in different cell types? Does this suggest similar or distinct functional roles in different cell types?

Minor comments:

1) 3rd line of the abstract: change “a regulator of neurogenesis by recruiting corepressor proteins” to “a regulator of neurogenesis that acts by recruiting corepressor proteins”

2) Line 6 of the abstract: change “evidence has shown the proteins have the ability to repress unique target gene expression” to “evidence has shown that these proteins have the ability to repress expression of distinct target genes”

3) Line 3 of the significance statement: change “specification and maturation of neurons” to “specification and maturation of neurons and glia"

4) 6th line of the significance statement: change “been shown to target unique genes, have distinct expression patterns and important functions” to “been shown to independently target unique genes, have distinct expression patterns, and important REST-independent functions”

5) The last sentence of the significance statement doesn’t make sense without further explanation - why would understanding transcriptional regulators that play a crucial role in neurodevelopment be relevant for understanding the basis of neurodegenerative disease, rather that, for example, neurodevelopmental disorders. The authors discuss later in the manuscript that evidence has shown that misregulation of REST-CoREST may contribute to neurodegeneration.......but without first presenting this information, the claim that understanding CoREST proteins is going to advance our understanding of neurodegeneration seems out of place.

6) In the first paragraph of section 2 the following sentence is confusing: “Chromatin immunoprecipitation-coupled with deep sequencing (ChIP-seq) experiments have identified the RE1 site to overlap with approximately 2000 genes within the human genom (Satoh et al., 2013; Rockowitz and Zheng,2015) and specifically 308 genes in neurons derived from human embryonic stem cells (ESCs)(Satoh et al., 2013).” Did sequencing experiments identify putative REST-binding sites (RE1 sites) in these genes or was REST found to be bound to 2000 and 308 genes?

7) Section 2.1, third paragraph: change “HDAC1/2 then deacetylate H3K9” to “HDAC1/2 then deacetylates H3K9”

8) 4th line of section 3: citations should be provided for the sentence “evidence suggests each of the CoREST family members elicits unique functions at different stages of development.”

9) Section 3.2 line 3: change “CoREST protein family elicit” to “CoREST proteins elicit”

10) Section 3.2, the last sentence of the second paragraph is confusing “Therefore epigenetic regulation of the complex......”

11) Section 3.3 third paragraph is a comparison and contrast between the different CoREST proteins. As it is currently written, it is a bit hard to follow. We may not need all this detailed information - what we need is more information about the potential functional impact of each of these proteins.

12) Section 3.3. would be easier to follow if more cleanly organized into: a paragraph about expression of CoREST across different brain regions (mRNA and protein data), a paragraph about expression of CoREST in different cell types (mRNA and protein data) and a paragraph about expression of CoREST at different stages of cell division. Currently these topics are partially intermixed making the information and its implications harder to synthesize. It would be helpful to spend more time elaborating on the functional implications of all these different expression patterns. For example, what do the different subcellular localizations of CoREST (cytosol versus nucleus) at different stages of cell division suggest about the specific regulatory role that CoREST might be playing in cell division or maturation?

13) Line 7 of section 3.4: modify the sentence to read “Through ChIP-on-chip analysis, a total of 1820 genes were found to be targets of CoREST1 and only 322 of these were bound to by REST in mouse NSCs (Abrajano et al., 2010).” Unless the 322 genes bound by REST were distinct genes from the 1820 genes targeted by CoREST, in which case the sentence needs to be worded in a way to make this clear.

14) Section 3.5 line 6: “facilitate gene repression” could be misunderstood. “contribute to gene repression” is more clear.

## Author Response

We thank the reviewers for their helpful and positive comments on the manuscript.

Reviewer #1:

Q1. This is a well-researched review paper that highlights a gap in knowledge of the independent functions of the CoREST protein family. Table 1 specifically is a very helpful summary of major findings from this review. In the introduction, there is an over-reliance on citing other review articles instead of the primary research. However, this is not the case in the rest of the paper.

Ans: Review citations have been removed and primary literature has been cited (page 2).

Through repressing the expression of target genes, REST regulates neurogenesis, neuronal differentiation and maturation (Ballas et al., 2005, Paquette et al., 2000, Gupta et al., 2009, Gao et al., 2011, Mandel et al., 2011), in addition to a playing role in neuroprotection (Lu et al., 2014, Suo et al., 2015, Song et al., 2016, Song et al., 2017b). Dysfunction of REST and its corepressor proteins are hypothesised to cause disruption in gene regulatory networks, contributing to the pathophysiology of neurodegenerative conditions, including Alzheimer's (Ashton et al., 2017, Lu et al., 2014, Meyer et al., 2019), Huntington's (Zuccato et al., 2003, Zuccato et al., 2007, Conforti et al., 2013), Parkinson's (Suo et al., 2015, Kawamura et al., 2019) and Prion disease (Song et al., 2017a, Song et al., 2016, Song et al., 2017b).

Q2. The introduction to section 3 has no citations, although, it mentions evidence, animal models, and cell models used to study the effects of CoREST dysfunction. It can be assumed the information provided there is broad and will be expounded later in the section with citations, however, a few substantiating citations may be beneficial.

Ans: References have now been added as follows (page 5):

“To date, several different models, including rodent (Monaghan et al., 2017, Wang et al., 2016), stem cell (Yang et al., 2011) and established cell lines (Gómez et al., 2008), have been utilised to study the role of CoREST proteins in development.”

Q3. There are some minor grammatical errors in section 4 lines 5-6 with incorrect or missing words. Overall, this is a good review of the functions of the CoREST proteins in neurodevelopment.

Ans: Changes have been made as requested, see below (page 12).

“For example in Huntington's disease (HD) the accumulation of nuclear REST in medium spiny neurons of the striatum results in enhanced repression of BDNF transcription contributing to an increased susceptibility to neuronal loss (Zuccato et al., 2003).”

Reviewer #2

Q1. The title leads us to believe that the meat of the review will be about functional roles of CoREST proteins in neurodevelopment, and more specifically, potential REST-independent functional roles of CoREST proteins. But the functional roles of REST and CoREST actually get relatively little treatment and elaboration compared to information about their expression patterns and composition of repressive complexes that they form etc.

a. In the introduction, functional roles of REST are summarized (“Through repressing the expression of target genes, REST regulates neurogenesis, neuronal differentiation and maturation (as reviewed in Ooi and Wood, 2007), in addition to a playing role in neuroprotection (as reviewed in Zhao et al. 2017). Dysfunction of REST and its corepressor proteins are hypothesised to cause disruption in gene regulatory networks, contributing to the pathophysiology of neurodegenerative conditions (as reviewed in Mampay and Sheridan 2019).” But no additional information is provided about the evidence for such functional roles and whether similar mechanisms are at operation in all these different contexts. In addition to citing the reviews, the authors should give us some summary of evidence suggesting these functions and the mechanisms involved. Is the mechanism exclusively transcriptional repression? Are different sets of genes targeted in each of these contexts and, if so, how is such specificity thought to be achieved?

Ans: The Introduction is a general overview of the review and therefore we believe it is not appropriate to go into specific details about the functional roles of REST and mechanisms involved in this section. Instead we have now provided this additional information within the section titled ‘2.1 Role of REST in neurogenesis, neuroprotection and neurodegeneration’. We have also changed the references to mention the primary source of the studies rather than general reviews.

Q2. Second paragraph in section 2, the authors say that REST represses transcription of genes associated with neuronal differentiation and maturation. But what are the consequences of this? That REST acts overall to promote maintenance of progenitor cell status? That REST regulates the timing of switches between neurogenesis and gliogenesis? That REST regulates the speed and timing of neuronal differentiation after cells have been specified?

Ans: We have now expanded this section to include additional information describing REST target genes and associated mechanisms of how neurogenesis and functions in mature neurons are regulated (Page 3).

Q3. Second paragraph in section 2: the authors tell us that REST and its co repressors have been shown to regulate neurogenesis, shape neuroprotection, and contribute to cancer and neurodenegerative disease when aberrantly expressed. But again they don't discuss at all the evidence for these proposed functional roles and the potential mechanisms at work here. This is critical if we want to go on to decide whether CoREST proteins independently exert functional roles that are similar to or distinct from those that occur in concert with REST.

Ans: We have now included additional information describing how REST may regulate neurogenesis, neuroprotection and neurodegeneration (page 3-4).

Q4. Section 3.5 “Functional roles of the CoREST family during neurodevelopment,” what should be the meat of the review, is packaged into a table. This should be the longest section of the review with a careful analysis of what exactly has been done to suggest that CoREST proteins can shape development independently of REST. What experimental approaches were used? What were the implications of these studies? What are the limitations of these studies and what is needed to advance our understanding of REST-independent CoREST function? Do we need specific mouse models that haven't been made for example? The authors say in a number of places that more research is needed, but it would be helpful for them to indicate what are THE most critical gaps in our knowledge at this point and what would be key experiments that could fill these gaps. It's fine to have a table that summarizes key studies, but this should NOT take the place of a careful analysis and discussion of current evidence and gaps in the literature.

Ans: We have now expanded this section to describe specific studies showing the role of CoREST proteins in neurodevelopment as well as discussing the critical gaps that need to be addressed in future studies.

Q5. Last two sentences of section 2.1 indicate that the stepwise activity of the REST complex influences neurogenesis, synaptic plasticity, and neuroprotection and that disruptions of this process contribute to neurodegenerative conditions. Again, since the title, significance statement and introduction indicate that we will be learning about the functions of REST and CoREST, it would be helpful to elaborate here on the what the evidence is for involvement in each of these functions. Is the same stepwise activity of the REST complex required for each of these functional roles? Are the target binding sites and genes the same or different for each of these proposed functional roles?

Ans: The role of REST has been extensively reviewed whilst there remains to be very few reviews focussed on the CoREST family. For this reason we have made the focus of this article the role of CoREST and minimised discussion about the role of REST that has been thoroughly described in the literature including (Mampay and Sheridan, 2019), (Hwang and Zukin, 2018), (Zhao et al., 2017), (Song et al., 2015),(Ooi and Wood, 2007) and (Ballas and Mandel, 2005).

The significance statement and introduction have been edited to make it clear that the focus of this review is CoREST. Nevertheless, this is an important area and we will address each point in brief in text.

Q6. Section 3.1 goes into great detail about how CoREST proteins recruit other players to the complex. But if we want to learn more about potential roles for CoREST proteins independent of REST, we need different / additional information. What mediates the interactions of CoREST with REST and what potential mechanisms would determine when this interaction occurs and under what circumstances this interaction doesn't occur? When CoREST proteins are acting independenty of REST - do they still need the interactions with all the other complex members to function?

Ans: As CoREST-mediated gene repression is an emerging field there has been limited research into the comparison of the presence of the REST complex vs the CoREST complex. Piloto et al., 2015 and Wu et al., 2018 were the two pioneer papers that detailed a potential mechanism for how CoREST complex mediates histone modifications and thus transcriptional repression. These are described in detail in section 3.2 (page 7).

Section 3.1 has been modified to include information on how CoREST interacts with REST (page 6). See below:

“CoREST proteins interact with the REST complex via a single zinc finger domain in the C-terminal half of REST (Andrés et al., 1999). A single point mutation resulted in abolished CoREST binding and transcriptional repression by the complex (Andrés et al., 1999). The function of the REST complex has been widely studied since discovery in 1995, with less known about the CoREST complex. Bioinformatics, structural analysis and immunoprecipitation assays of the CoREST family has provided insight into the components of the complex, how it interacts with DNA and potential mechanisms of epigenetic modifications to regulate gene expression.”

In addition, Section 3.4 has been edited to include additional information on ChIP-seq data for each CoREST protein (page 10).

“Genome wide ChIP-seq in mouse ESCs investigating the binding of REST and its cofactors to sites on the genome identified CoREST1 to have 84 peaks with 61 overlapping with REST, CoREST2 to have 459 genomic binding sites and only 43 overlap with REST and CoREST3 to have 3744 peaks and 885 overlap with REST (Yu et al., 2011). Further work is required to identify whether the CoREST proteins binding directly to DNA, the cofactors recruited to the genomic binding site and whether the genes are functionally repressed by the activity of the complex.”

Q7. Section 3.2 - emphasize that what is being presented is the mechanism by which CoREST proteins are thought to exert their REST-independent activity. Help the reader who may not be directly in the field by drawing some comparisons to the most important distinctions between REST-CoREST mediated transcriptional repression and CoREST mediated transcriptional repression. The Sant2 domain is mentioned as mediating DNA binding - is this absolutely necessary for CoREST LCH complex action on DNA independent of REST? What happens if this domain is mutated in CoREST proteins? What genes contain binding sites for this Sant2 domain and does this account for different genes being targeted by REST-CoREST versus CoREST LCH compex? How do REST-CoREST and CoREST LCH complex transcriptional repression compare in terms of potency and permanence?

Ans: There is limited research investigating genes that contain binding sites for SANT2 and the differences in the targets and potency of the REST-CoREST and LCH complex which poses a void in the current literature.

The final sentences of section 3.2 have been edited to indicate these questions are still to be answered in the field. See below (page 8).

“There is a prominent void in the literature regarding the differences in transcriptional repression potency and activity between the REST-CoREST and the LCH complex. Further research is required to confirm the formation of the LCH complex in vivo, characterise the DNA sequence at the binding site of the complex and thereby identify the gene targets for each CoREST protein.”

Q8. When discussing the various genes targeted by REST and CoREST in section 3.4, it's not clear how much overlap there is in the genes targeted by each. Are the genes targeted by REST just a subset of those targeted by CoREST or are they completely different genes? What are the broad functional families into which REST versus CoREST alone targeted genes? What does this suggest about functional roles of REST-CoREST complex versus the CoREST LCH complex? How much overlap is there in the genes targeted by CoREST in different cell types? Does this suggest similar or distinct functional roles in different cell types?

Ans: Section 3.4 has now been refined and expanded to address these questions.

Q9. 3rd line of the abstract: change “a regulator of neurogenesis by recruiting corepressor proteins” to “a regulator of neurogenesis that acts by recruiting corepressor proteins”

Ans: Manuscript has been edited as requested.

Q10. Line 6 of the abstract: change “evidence has shown the proteins have the ability to repress unique target gene expression” to “evidence has shown that these proteins have the ability to repress expression of distinct target genes”

Ans: Manuscript has been edited as requested.

Q11. Line 3 of the significance statement: change “specification and maturation of neurons” to “specification and maturation of neurons and glia”

Ans: Manuscript has been edited as requested.

Q12. 6th line of the significance statement: change “been shown to target unique genes, have distinct expression patterns and important functions” to “been shown to independently target unique genes, have distinct expression patterns, and important REST-independent functions”

Ans: Manuscript has been edited as requested.

Q13. The last sentence of the significance statement doesn't make sense without further explanation - why would understanding transcriptional regulators that play a crucial role in neurodevelopment be relevant for understanding the basis of neurodegenerative disease, rather that, for example, neurodevelopmental disorders. The authors discuss later in the manuscript that evidence has shown that misregulation of REST-CoREST may contribute to neurodegeneration.......but without first presenting this information, the claim that understanding CoREST proteins is going to advance our understanding of neurodegeneration seems out of place.

Ans: We have now modified this sentence to “Understanding the molecular mechanisms governed by the CoREST family will provide insight into the regulatory networks directing normal neurodevelopment.”

Q14. In the first paragraph of section 2 the following sentence is confusing: “Chromatin immunoprecipitation-coupled with deep sequencing (ChIP-seq) experiments have identified the RE1 site to overlap with approximately 2000 genes within the human genom (Satoh et al., 2013; Rockowitz and Zheng,2015) and specifically 308 genes in neurons derived from human embryonic stem cells (ESCs)(Satoh et al., 2013).” Did sequencing experiments identify putative REST-binding sites (RE1 sites) in these genes or was REST found to be bound to 2000 and 308 genes?

Ans: We have now clarified this statement: “Chromatin immunoprecipitation-coupled with deep sequencing (ChIP-seq) experiments have identified REST to bind with approximately 2000 genes within the human genome (Rockowitz and Zheng, 2015, Satoh et al., 2013) and 308 genes in neurons derived from human embryonic stem cells (ESCs) unique to the targets observed in ESCs (Satoh et al., 2013).”

Q15. Section 2.1, third paragraph: change “HDAC1/2 then deacetylate H3K9” to “HDAC1/2 then deacetylates H3K9”

Ans: Edited to “HDAC1/2 then deacetylates H3K9”

Q16. 4th line of section 3: citations should be provided for the sentence “evidence suggests each of the CoREST family members elicits unique functions at different stages of development.”

Ans: We have now provided references to support this statement: “evidence suggests each of the CoREST family members elicits unique functions at different stages of development (Wang et al., 2016, Jung et al., 2018, Yang et al., 2011).”

Q17. Section 3.2 line 3: change “CoREST protein family elicit” to “CoREST proteins elicit” Ans: Edited as requested.

Q18. Section 3.2, the last sentence of the second paragraph is confusing “Therefore epigenetic regulation of the complex......”

Ans: Edited to “Therefore, epigenetic regulation mediated by the LCH complex will be reduced when chromatin is marked by acetylation at Lys14,”

Q19. Section 3.3 third paragraph is a comparison and contrast between the different CoREST proteins. As it is currently written, it is a bit hard to follow. We may not need all this detailed information - what we need is more information about the potential functional impact of each of these proteins.

Ans: Please see answer to Question 20.

Q20. Section 3.3. would be easier to follow if more cleanly organized into: a paragraph about expression of CoREST across different brain regions (mRNA and protein data), a paragraph about expression of CoREST in different cell types (mRNA and protein data) and a paragraph about expression of CoREST at different stages of cell division. Currently these topics are partially intermixed making the information and its implications harder to synthesize. It would be helpful to spend more time elaborating on the functional implications of all these different expression patterns. For example, what do the different subcellular localizations of CoREST (cytosol versus nucleus) at different stages of cell division suggest about the specific regulatory role that CoREST might be playing in cell division or maturation?

Ans for Q19 and Q20: This section has been broken into three subsections as requested and additional information was included to describe the significance of the expression pattern/localisation in regards to function. The additional subsections are:

“3.3.1 CoREST paralogues exhibit an age-dependent and region specific expression pattern in the brain;

3.3.2 Differential subcellular localisation of the CoREST family in different cell types;

3.3.3 CoREST2 expression changes throughout cell division”

Q21. Line 7 of section 3.4: modify the sentence to read “Through ChIP-on-chip analysis, a total of 1820 genes were found to be targets of CoREST1 and only 322 of these were bound to by REST in mouse NSCs (Abrajano et al., 2010).” Unless the 322 genes bound by REST were distinct genes from the 1820 genes targeted by CoREST, in which case the sentence needs to be worded in a way to make this clear.

Ans: The sentence has now been edited as follows “Through ChIP-on-chip analysis in mouse NSCs, CoREST1 was shown to bind to a broader range of genes (1820 genes) compared to REST (322 genes)(Abrajano et al., 2010). Of these genes only 126 were targets of both REST and CoREST1. CoREST1 was identified to target a significantly greater percentage of genes involved in pluripotency such as NANOG/OCT4/SOX2 network compared to REST (Abrajano et al., 2010). Suggesting CoREST1 has a widespread role in regulating NSC gene networks that is unique to REST.”

Q22. Section 3.5 line 6: “facilitate gene repression” could be misunderstood. “contribute to gene repression” is more clear.

Ans: Edited as requested.

References

ABRAJANO, J. J., QURESHI, I. A., GOKHAN, S., MOLERO, A. E., ZHENG, D., BERGMAN, A. & MEHLER, M. F. 2010. Corepressor for element-1-silencing transcription factor preferentially mediates gene networks underlying neural stem cell fate decisions. Proceedings of the National Academy of Sciences of the United States of America, 107, 16685-16690.

ASHTON, N. J., HYE, A., LECKEY, C. A., JONES, A. R., GARDNER, A., ELLIOTT, C., WETHERELL, J. L., LENZE, E. J., KILLICK, R. & MARCHANT, N. L. 2017. Plasma REST: a novel candidate biomarker of Alzheimer's disease is modified by psychological intervention in an at-risk population. Translational Psychiatry, 7, e1148.

BALLAS, N., GRUNSEICH, C., LU, D. D., SPEH, J. C. & MANDEL, G. 2005. REST and Its Corepressors Mediate Plasticity of Neuronal Gene Chromatin throughout Neurogenesis. Cell, 121, 645-65.7

BALLAS, N. & MANDEL, G. 2005. The many faces of REST oversee epigenetic programming of neuronal genes. Current Opinion in Neurobiology, 15, 500-506.

CONFORTI, P., ZUCCATO, C., GAUDENZI, G., IERACI, A., CAMNASIO, S., BUCKLEY, N. J., MUTTI, C., COTELLI, F., CONTINI, A. & CATTANEO, E. 2013. Binding of the repressor complex REST-mSIN3b by small molecules restores neuronal gene transcription in Huntington's disease models. Journal of Neurochemistry, 127, 22-35.

GAO, Z., URE, K., DING, P., NASHAAT, M., YUAN, L., MA, J., HAMMER, R. E. & HSIEH, J. 2011. The Master Negative Regulator REST/NRSF Controls Adult Neurogenesis by Restraining the Neurogenic Program in Quiescent Stem Cells. The Journal of Neuroscience, 31, 9772-9786.

GÓMEZ, A. V., GALLEGUILLOS, D., MAASS, J. C., BATTAGLIOLI, E., KUKULJAN, M. & ANDRÉS, M. E. 2008. CoREST represses the heat shock response mediated by HSF1. Molecular Cell, 31, 222-231.

GUPTA, S. K., GRESSENS, P. & MANI, S. 2009. NRSF downregulation induces neuronal differentiation in mouse embryonic stem cells. Differentiation, 77, 19-28.

HWANG, J.-Y. & ZUKIN, R. S. 2018. REST, a master transcriptional regulator in neurodegenerative disease. Current opinion in neurobiology, 48, 193-200.

JUNG, H. G., HWANG, Y. S., PARK, Y. H., CHO, H. Y., RENGARAJ, D. & HAN, J. Y. 2018. Role of Epigenetic Regulation by the REST/CoREST/HDAC Corepressor Complex of Moderate NANOG Expression in Chicken Primordial Germ Cells. Stem Cells and Development, 27, 1215-1225.

KAWAMURA, M., SATO, S., MATSUMOTO, G., FUKUDA, T., SHIBA-FUKUSHIMA, K., NODA, S., TAKANASHI, M., MORI, N. & HATTORI, N. 2019. Loss of nuclear REST/NRSF in aged-dopaminergic neurons in Parkinson's disease patients. Neuroscience Letters, 699, 59-63.

LU, T., ARON, L., ZULLO, J., PAN, Y., KIM, H., CHEN, Y., YANG, T.-H., KIM, H.-M., DRAKE, D., LIU, X. S., BENNETT, D. A., COLAIÁCOVO, M. P. & YANKNER, B. A. 2014. REST and stress resistance in ageing and Alzheimer's disease. Nature, 507, 448.

MAMPAY, M. & SHERIDAN, G. K. 2019. REST: An epigenetic regulator of neuronal stress responses in the young and ageing brain. Frontiers in Neuroendocrinology, 53, 100744.

MANDEL, G., FIONDELLA, C. G., COVEY, M. V., LU, D. D., LOTURCO, J. J. & BALLAS, N. 2011. Repressor element 1 silencing transcription factor (REST) controls radial migration and temporal neuronal specification during neocortical development. Proceedings of the National Academy of Sciences, 108, 16789-16794.

MEYER, K., FELDMAN, H. M., LU, T., DRAKE, D., LIM, E. T., LING, K.-H., BISHOP, N. A., PAN, Y., SEO, J., LIN, Y.-T., SU, S. C., CHURCH, G. M., TSAI, L.-H. & YANKNER, B. A. 2019. REST and Neural Gene Network Dysregulation in iPSC Models of Alzheimer's Disease. Cell Reports, 26, 1112-1127.e9.

MONAGHAN, C. E., NECHIPORUK, T., JENG, S., MCWEENEY, S. K., WANG, J., ROSENFELD, M. G. & MANDEL, G. 2017. REST corepressors RCOR1 and RCOR2 and the repressor INSM1 regulate the proliferation-differentiation balance in the developing brain. Proceedings of the National Academy of Sciences of the United States of America, 114, E406-E415.

OOI, L. & WOOD, I. C. 2007. Chromatin crosstalk in development and disease: lessons from REST. Nature Reviews Genetics, 8, 544.

PAQUETTE, A. J., PEREZ, S. E. & ANDERSON, D. J. 2000. Constitutive expression of the neuron-restrictive silencer factor (NRSF)/REST in differentiating neurons disrupts neuronal gene expression and causes axon pathfinding errors in vivo. Proceedings of the National Academy of Sciences, 97, 12318-12323.

ROCKOWITZ, S. & ZHENG, D. 2015. Significant expansion of the REST/NRSF cistrome in human versus mouse embryonic stem cells: potential implications for neural development. Nucleic Acids Research, 43, 5730-5743.

SATOH, J.-I., KAWANA, N. & YAMAMOTO, Y. 2013. ChIP-Seq Data Mining: Remarkable Differences in NRSF/REST Target Genes between Human ESC and ESC-Derived Neurons. Bioinformatics and Biology Insights, 7, 357-368.

SONG, Z., SHAH, S. Z. A., YANG, W., DONG, H., YANG, L., ZHOU, X. & ZHAO, D. 2017a. Downregulation of the Repressor Element 1-Silencing Transcription Factor (REST) Is Associated with Akt-mTOR and Wnt-β-Catenin Signaling in Prion Diseases Models. Frontiers in Molecular Neuroscience, 10.

SONG, Z., YANG, W., ZHOU, X., YANG, L. & ZHAO, D. 2017b. Lithium alleviates neurotoxic prion peptide-induced synaptic damage and neuronal death partially by the upregulation of nuclear target REST and the restoration of Wnt signaling. Neuropharmacology, 123, 332-348.

SONG, Z., ZHAO, D., ZHAO, H. & YANG, L. 2015. NRSF: an Angel or a Devil in Neurogenesis and Neurological Diseases. Journal of Molecular Neuroscience, 56, 131-144.

SONG, Z., ZHU, T., ZHOU, X., BARROW, P., YANG, W., CUI, Y., YANG, L. & ZHAO, D. 2016. REST alleviates neurotoxic prion peptide-induced synaptic abnormalities, neurofibrillary degeneration and neuronal death partially via LRP6-mediated Wnt-β-catenin signaling. Oncotarget, 7, 12035-12052.

SUO, H., WANG, P., TONG, J., CAI, L., LIU, J., HUANG, D., HUANG, L., WANG, Z., HUANG, Y., XU, J., MA, Y., YU, M., FEI, J. & HUANG, F. 2015. NRSF is an essential mediator for the neuroprotection of trichostatin A in the MPTP mouse model of Parkinson's disease. Neuropharmacology, 99, 67-78.

WANG, Y., WU, Q., YANG, P., WANG, C., LIU, J., DING, W., LIU, W., BAI, Y., YANG, Y., WANG, H., GAO, S. & WANG, X. 2016. LSD1 co-repressor Rcor2 orchestrates neurogenesis in the developing mouse brain. Nature Communications, 7, 10481.

YANG, P., WANG, Y., CHEN, J., LI, H., KANG, L., ZHANG, Y., CHEN, S., ZHU, B. & GAO, S. 2011. RCOR2 is a subunit of the LSD1 complex that regulates ESC property and substitutes for SOX2 in reprogramming somatic cells to pluripotency. Stem Cells, 29, 791-801.

YU, H.-B., JOHNSON, R., KUNARSO, G. & STANTON, L. W. 2011. Coassembly of REST and its cofactors at sites of gene repression in embryonic stem cells. Genome research, 21, 1284-1293.

ZHAO, Y., ZHU, M., YU, Y., QIU, L., ZHANG, Y., HE, L. & ZHANG, J. 2017. Brain REST/NRSF Is Not Only a Silent Repressor but Also an Active Protector. Molecular Neurobiology, 54, 541-550.

ZUCCATO, C., BELYAEV, N., CONFORTI, P., OOI, L., TARTARI, M., PAPADIMOU, E., MACDONALD, M., FOSSALE, E., ZEITLIN, S., BUCKLEY, N. & CATTANEO, E. 2007. Widespread Disruption of Repressor Element-1 Silencing Transcription Factor/Neuron-Restrictive Silencer Factor Occupancy at Its Target Genes in Huntington's Disease. The Journal of Neuroscience, 27, 6972-6983.

ZUCCATO, C., TARTARI, M., CROTTI, A., GOFFREDO, D., VALENZA, M., CONTI, L., CATAUDELLA, T., LEAVITT, B. R., HAYDEN, M. R., TIMMUSK, T., RIGAMONTI, D. & CATTANEO, E. 2003. Huntingtin interacts with REST/NRSF to modulate the transcription of NRSE-controlled neuronal genes. Nature Genetics, 35, 76.
